# Application of pedicle bone quality scoring and Hounsfield unit in predicting the risk of cage subsidence after single-segment lumbar fusion surgery

**DOI:** 10.3389/fmed.2026.1669642

**Published:** 2026-02-18

**Authors:** Jinxiang Zhan, Shiji Chen, Qipeng Wei, Zihao Liu, Weijun Guo, Qingyan Huang, Dongling Cai

**Affiliations:** 1 Department of Orthopedics, Panyu Hospital of Chinese Medicine, Guangzhou, Guangdong, China; 2 Guangzhou University of Chinese Medicine, Guangzhou, Guangdong, China

**Keywords:** cage subsidence, Hounsfield units, pedicle bone quality scoring, predictive model, risk factor analysis

## Abstract

**Objective:**

This study aimed to investigate the clinical value of combining pedicle bone quality scoring and Hounsfield units (HUs) in predicting the risk of cage subsidence following single-segment lumbar fusion surgery.

**Methods:**

We conducted a retrospective analysis of clinical data from 160 patients who underwent single-segment lumbar fusion surgery at Panyu District Traditional Chinese Medicine Hospital between January 2017 and May 2023. Clinical data comparisons and multivariate logistic regression analyses were performed using SPSS 27.0. Receiver operating characteristic (ROC) curves were generated using MedCalc 23 to evaluate diagnostic efficacy.

**Results:**

Comparative analysis of clinical data revealed statistically significant differences between the two groups in terms of age, hypertension, HUs, vertebral body quality (VBQ) scores, and pedicle bone quality (PBQ) scores (*P* < 0.05). The multivariate logistic regression analysis indicated that HUs (OR = 0.98, 95% CI: 0.97–0.99; *P* = 0.018) and PBQ scores (OR = 3.99, 95% CI: 1.63–9.79; *P* = 0.002) are independent predictors of cage subsidence in patients undergoing single-segment lumbar interbody fusion. ROC analysis demonstrated that the area under the curve (AUC) for HUs was 0.781 (95% CI: 0.709–0.843), with an optimal threshold of 95, yielding a maximum Youden index of 0.460, corresponding to a diagnostic sensitivity of 60.0% and specificity of 86.0%. The AUC for PBQ scores was 0.772 (95% CI: 0.699–0.835), with an optimal threshold of 3.088, yielding a maximum Youden index of 0.480, corresponding to a diagnostic sensitivity of 85.0% and specificity of 63.0%. Furthermore, the predictive model constructed by combining HUs and PBQ scores achieved an AUC of 0.831 (95% CI: 0.763–0.885), with a maximum Youden index of 0.557, resulting in a diagnostic sensitivity of 76.7% and specificity of 79.0%. DeLong’s test results indicated that the combined diagnostic model outperformed the individual use of HUs and PBQ scores (*P*-values of 0.049 and 0.014, respectively).

**Conclusion:**

Pedicle bone quality scoring and HUs provide significant reference value in diagnosing cage subsidence following single-segment lumbar interbody fusion. The predictive model constructed through the combined assessment of these two factors demonstrated superior diagnostic efficacy.

## 1 Introduction

Single-segment lumbar interbody fusion (LIF) is a commonly performed surgical procedure, primarily used to treat lumbar instability and degenerative diseases. It has been shown to effectively alleviate pain and improve functional outcomes in patients (1–3). However, despite the significant therapeutic effects, postoperative complications, particularly cage subsidence, remain key factors influencing both the immediate surgical outcome and long-term prognosis. cage subsidence can lead to postoperative low back pain, lower limb numbness and weakness, spinal deformities, and even severe issues such as nerve injury and fusion failure (4), all of which have a substantial impact on patients’ quality of life and the overall success of the surgery. Therefore, preventing and identifying the risk of cage subsidence is critical for optimizing treatment strategies.

In recent years, pedicle bone quality scoring (PBQ scoring) and Hounsfield Units (HUs) have emerged as essential tools for evaluating spinal bone quality and structure (5–7), garnering increased attention from researchers. PBQ scoring, developed based on the vertebral body quality (VBQ) scoring system, provides a novel method for assessing bone quality by quantifying the bone density and morphological characteristics of the pedicle (5, 8). Concurrently, HUs offer a direct imaging basis for evaluating structural changes and pathological conditions of the vertebral body (9). Existing studies have highlighted a strong correlation between the decline in bone quality and the occurrence of postoperative complications (10–12). However, there is still a lack of comprehensive research data regarding the specific role of PBQ scoring and HUs in predicting cage subsidence following single-segment lumbar fusion.

This study aimed to explore the clinical value of combining pedicle bone quality scoring and HUs in predicting the risk of cage subsidence after single-segment lumbar fusion. By analyzing clinical data from patients and establishing a multivariate logistic regression model, we aim to identify independent risk factors associated with cage subsidence and assess the potential clinical applicability of these indices. The findings of this study will provide scientific evidence to assist clinicians in preoperative risk assessment and the development of personalized treatment plans, ultimately improving the therapeutic outcomes and safety of single-segment lumbar fusion surgery.

## 2 Materials and methods

### 2.1 General information

This retrospective study analyzed the clinical data of 160 patients who were hospitalized in the Department of Orthopedics at Panyu District Traditional Chinese Medicine Hospital from January 2017 to May 2023. Postoperative follow-up was conducted for 18 months, during which lumbar X-ray or CT imaging was used to assess whether the implant had violated the endplate. cage subsidence was defined as penetration of the implant into any endplate by more than 2 mm. Based on the degree of cage subsidence, the 160 patients were divided into the cage subsidence group (*n* = 60) and the non-subsidence group (*n* = 100). This study was approved by the Ethics Committee of Panyu District Traditional Chinese Medicine Hospital. Given the retrospective nature of the study, the requirement for individual consent was waived. Demographic data collected included age, sex, history of hypertension, diabetes, smoking, steroid use, surgical segment, BMI, length of hospital stay, duration of surgery, and intraoperative blood loss. Radiological data included preoperative intervertebral space height, postoperative intervertebral space height, improvement in intervertebral space height, HUs, as well as VBQ and PBQ scores from T1-weighted MRI images.

#### 2.1.1 Inclusion criteria

(1) Patients underwent single-segment lumbar interbody fusion surgery at our hospital, and preoperative lumbar X-ray, CT, and MRI data were complete. (2) Follow-up duration exceeded 12 months, with follow-up data including lumbar X-ray or CT images. (3) Patients were aged over 40 years.

#### 2.1.2 Exclusion criteria

(1) Patients who had previously undergone lumbar fusion surgery. (2) Patients with ankylosing spondylitis, lumbar infections, lumbar tumors, or spinal trauma. (3) Patients who exhibited cage subsidence during the first follow-up after discharge.

### 2.2 Radiological data measurement

#### 2.2.1 Intervertebral space height measurement

**FIGURE 1:**
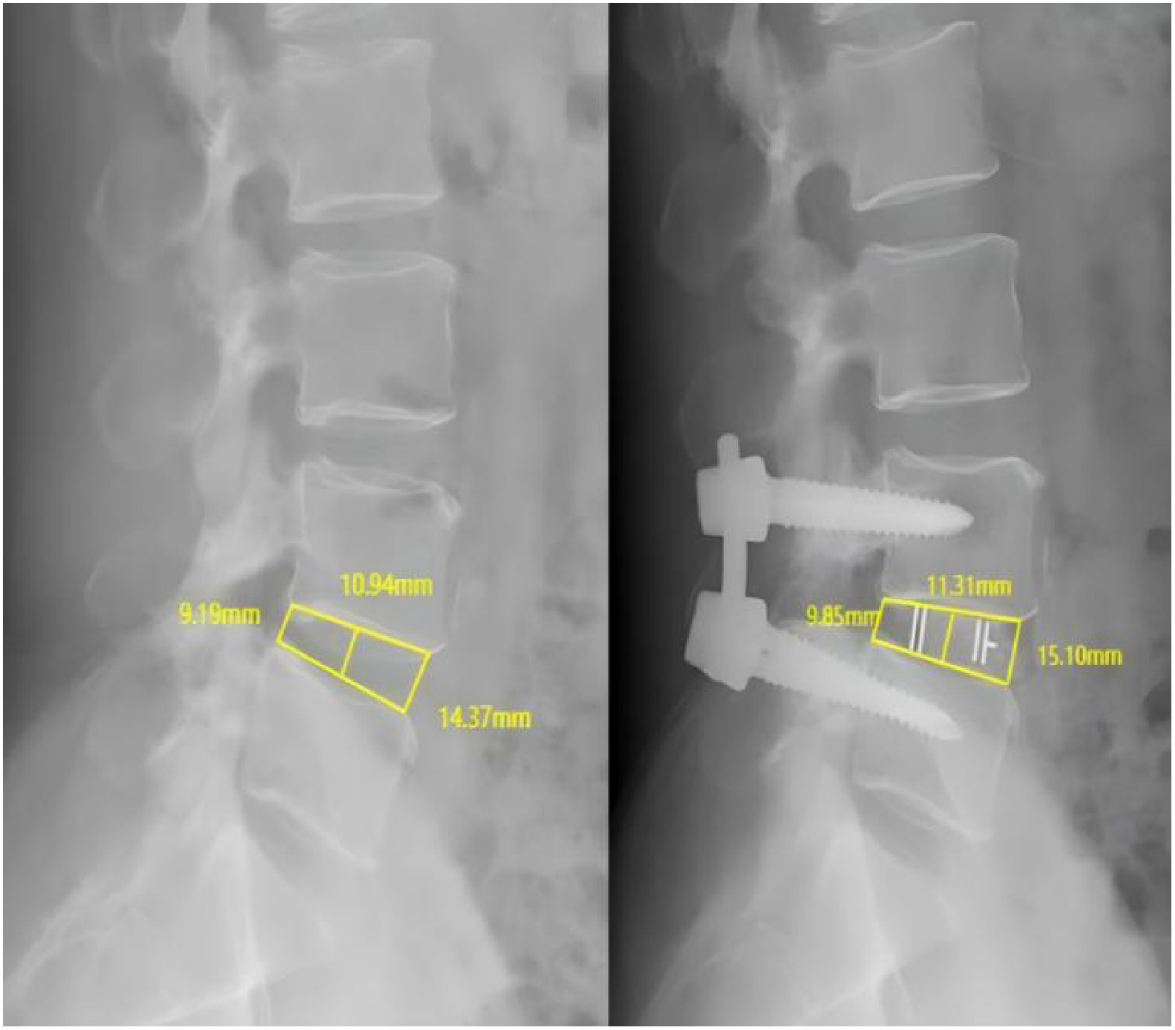
Preoperative and postoperative lumbar DR showing the measurement of the height of the anterior and posterior margins of the L4–5 intervertebral space.

Measurements were performed on standardized lateral lumbar radiographs with the patient in a standing position. The anterior point was defined as the most anterior margin of the vertebral endplate, the posterior point as the most posterior margin adjacent to the spinal canal, and the mid-point as the midpoint between the anterior and posterior edges. The PACS system’s built-in calibration tool, which uses the known size of the radiographic marker, was used to ensure accurate distance measurements. On lateral lumbar spine radiographs, intervertebral space height are assessed at the anterior, middle, and posterior edges of the intervertebral spaces. The average of these three measurements is defined as the height of the intervertebral space. The improvement in intervertebral space height is quantified by calculating the absolute difference between preoperative and postoperative intervertebral heights (Figure 1).

#### 2.2.2 HU Measurement

**FIGURE 2:**
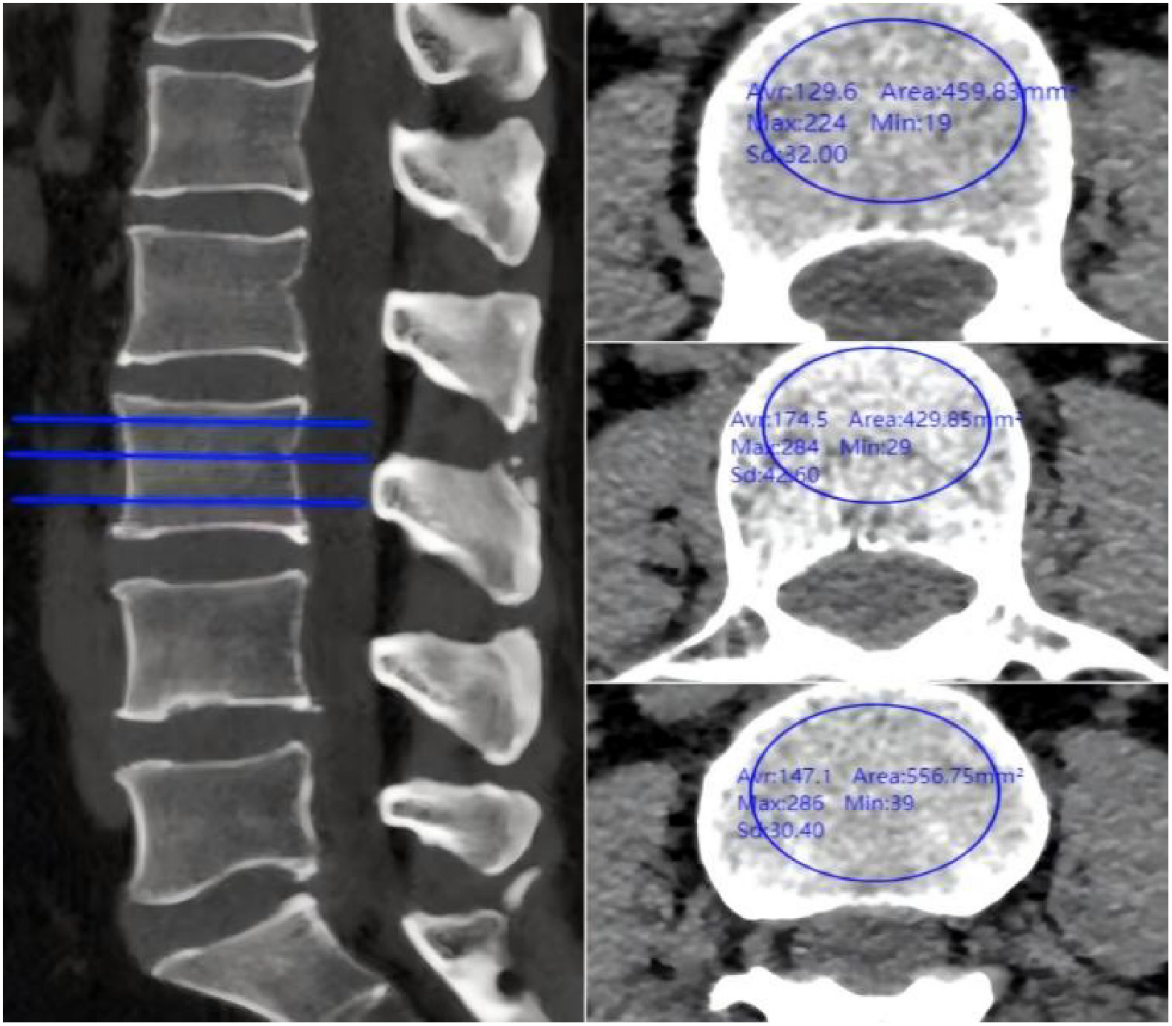
Preoperative lumbar CT showing individual vertebral HU measurements.

Imaging of the lumbar spine was performed using a Philips Brilliance 64-slice spiral CT scanner (tube voltage: 120 kV; tube current: 400–500 mA; reconstruction slice thickness: 1 mm; bone window width: 1,500 Hounsfield units (HU); window level: 500 HU). Radiographic measurements were conducted via the hospital PACS system. For CT imaging of the L1–L4 vertebrae, the focus was placed on the trabecular bone area at the center of the vertebral body. On axial CT slices, a standardized elliptical ROI with an area of approximately 150–200 mm^2^ was placed within the trabecular bone, carefully avoiding the cortical shell, basivertebral vein and any visible osteophytes or sclerotic areas. The PACS system automatically calculated the mean HU value for each vertebra (L1–L4). HUs were measured at three different levels for each vertebra: immediately below the superior endplate, at the mid-vertebral level, and immediately above the inferior endplate. The average HU for each vertebra was then calculated to obtain the overall HU (Figure 2).

#### 2.2.3 VBQ scoring measurement

**FIGURE 3:**
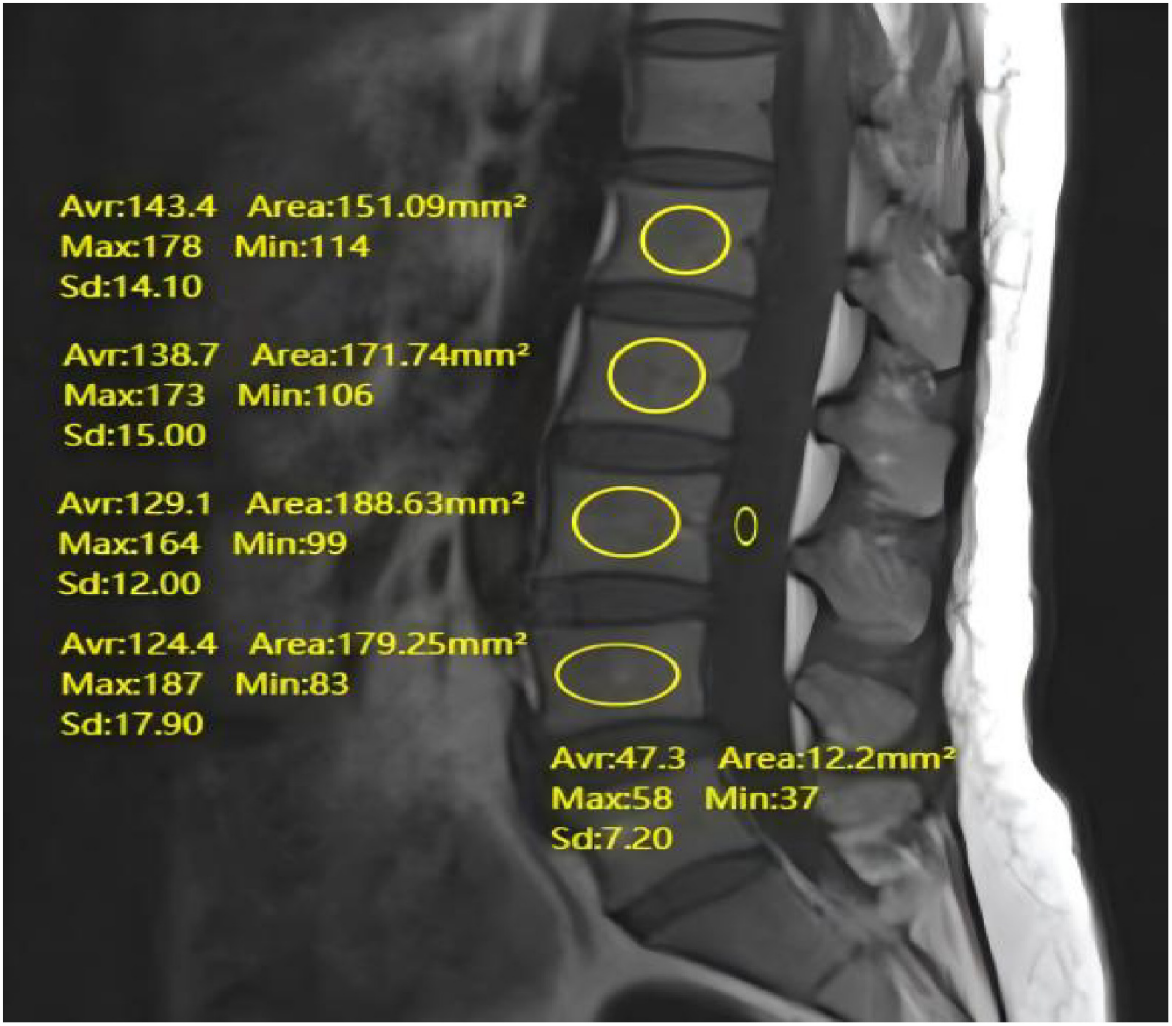
Preoperative lumbar MR demonstrating measurement of VBQ score.

Preoperative lumbar MRI scans were performed using a 1.5T Siemens system, covering L1–L4. A standard lumbar spine coil was used. T1-weighted imaging was performed using a fast spin-echo sequence with the following parameters: repetition time = 495 ms, echo time = 8 ms, slice thickness = 5 mm, slice gap = 1 mm, matrix = 192 × 235. Using the PACS system, measure the VBQ score on T1-weighted images. The ROI was set in the bone marrow area of the L1–L4 vertebrae and in the CSF area at the L3 level. Measure the average signal intensity within the ROI and compare it with the average signal intensity of the CSF at the L3 level. The VBQ score was then calculated by dividing the average signal intensity of the L1–L4 vertebrae by the CSF signal intensity at the L3 level (see Figure 3).

#### 2.2.4 PBQ scoring measurement

**FIGURE 4:**
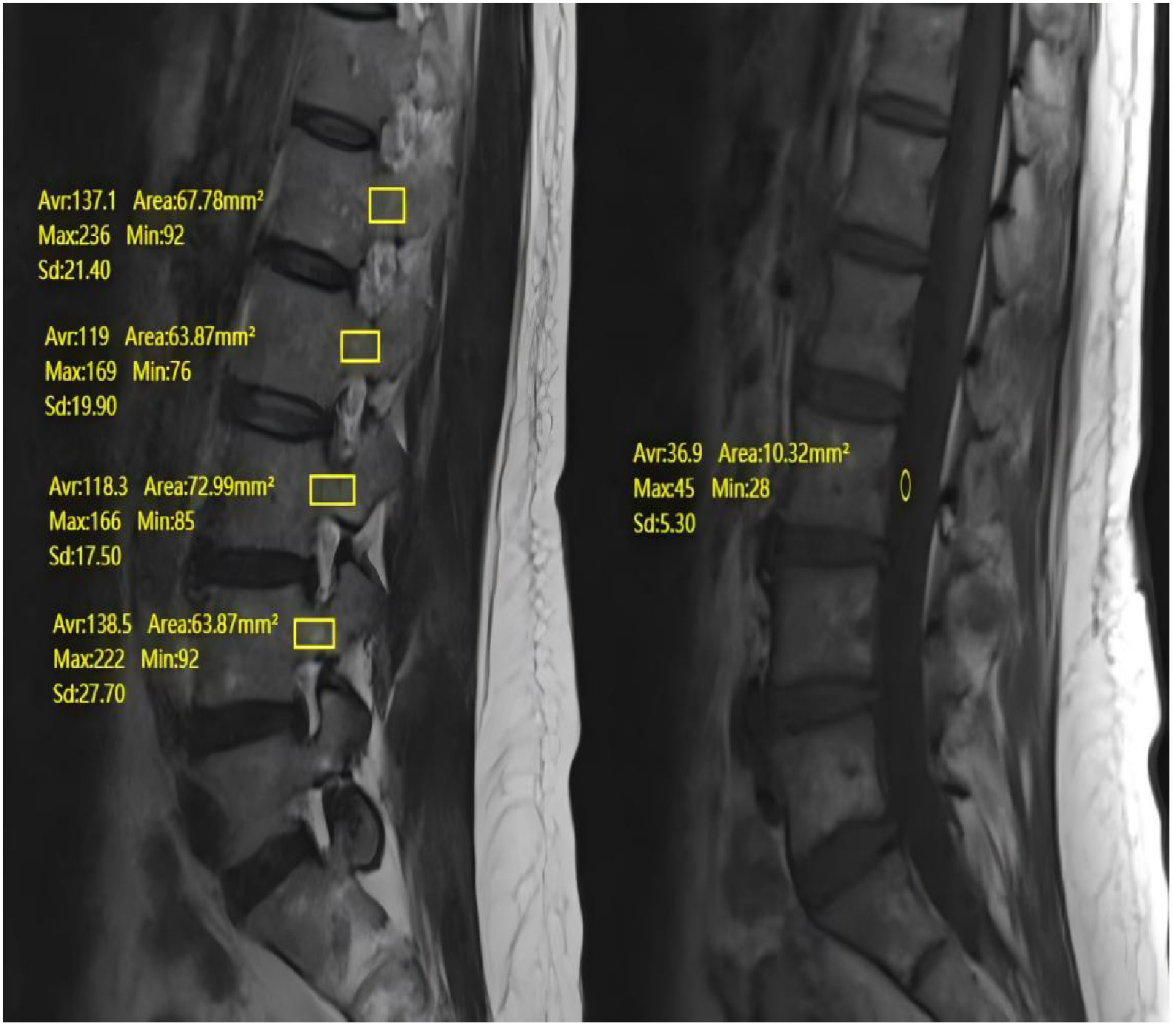
Preoperative lumbar MR showing measurement of PBQ score.

The PBQ score was calculated from routine lumbar spine MRI scans, including the L1–L4 vertebral bodies, using a 1.5T Siemens MRI system. A standard lumbar spine coil was used. T1-weighted imaging was performed using a fast spin-echo sequence with the following parameters: repetition time = 495 ms, echo time = 8 ms, slice thickness = 5 mm, slice gap = 1 mm, matrix = 192 × 235. The PBQ score was calculated using T1-weighted images via the PACS system. When doing so, carefully draw the ROI to follow the contour of the cancellous bone in the pedicle, maintaining a consistent distance of 1 mm from the inner edge of the cortical margin on all sequential slices where the pedicle is visible. If a focal lesion occupied more than 50% of the pedicle area on all visible slices, that particular pedicle was excluded from the analysis. If all sagittal slices were affected, the PBQ score was calculated based only on the unaffected slices, using the average pedicle value at those levels. The ROI for CSF was placed in an area of the posterior portion of the L3 vertebra that was free of descending nerve roots. If the CSF at the L3 level was affected, measurements were taken at the L2 or L4 levels instead. The PBQ score is calculated by dividing the median signal intensity (SI) of the L1–L4 pedicles by the CSF signal intensity at the L3 level. The final PBQ score reflects the average bone quality index derived from two pedicles (see Figure 4).

In this study, two experienced radiologists conducted all imaging data measurements and observations in a blinded manner to minimize potential biases, with their assessments performed independently. Both radiologists underwent standardized training prior to the study to ensure consistency, which included a review of the measurement protocol and radiological parameter assessment standards. The final data were obtained by averaging the measurements from both radiologists. Interobserver reliability was assessed using the intraclass correlation coefficient (ICC) with a two-way random-effects model for absolute agreement. The final data used for all subsequent analyses were the averages of the radiologists’ measurements.

### 2.3 Statistical analysis

Statistical analysis was performed using SPSS 27.0 software, with a significance level set at α = 0.05. A *p* < 0.05 was considered statistically significant. Initially, clinical data were compared: for continuous variables following a normal distribution, independent t-tests or analysis of variance (ANOVA) were used, and results were expressed as mean ± standard deviation (−x ± s). For non-normally distributed continuous variables, the appropriate non-parametric tests were applied, and the data were presented as median (25th percentile, 75th percentile), i.e., M(P25, P75). Categorical variables were analyzed using chi-square tests or Fisher’s exact test. Before performing multivariate logistic regression analysis, we assessed multicollinearity among the independent variables by calculating the variance inflation factor (VIF) and tolerance values. The results showed that the VIF values were well below 10, and all tolerance values were above 0.1, indicating no significant multicollinearity among the variables. Subsequently, multivariate logistic regression analysis was conducted, with the occurrence of cage subsidence as the dependent variable. Independent variables included gender, history of hypertension, HU, VBQ score, and PBQ score. Finally, receiver operating characteristic (ROC) curve analysis and DeLong’s test were performed using MedCalc software (version 23.0). The Youden index was calculated using the following formula: Youden Index = Sensitivity + Specificity − 1 (13).

## 3 Results

### 3.1 Comparison of clinical data

The comparison of baseline characteristics revealed statistically significant differences between the two groups in terms of age, hypertension, HU, VBQ score, and PBQ score (*P* < 0.05) (Table 1). However, no significant statistical differences were found between the groups regarding gender, diabetes, smoking history, steroid use, surgical segment, BMI, length of hospital stay, duration of surgery, intraoperative blood loss, preoperative intervertebral space height, postoperative intervertebral space height, or intervertebral space height change (*P* > 0.05) (Table 1).

**TABLE 1:** Comparison of clinical data between the cage subsidence group and the Non-cage subsidence group.

Variables	Total (*n* = 160)	Non-cage subsidence group (*n* = 100)	Cage subsidence group (*n* = 60)	Statistic	*P*
Age (years)	58.00 (53.00, 67.00)	56.50 (51.00, 63.00)	65.00 (56.00, 70.25)	*Z* = −3.92	<0.001
Gender				χ^2^ = 2.28	0.131
Female	120 (75.00)	71 (71.00)	49 (81.67)		
Male	40 (25.00)	29 (29.00)	11 (18.33)		
Hypertension				χ^2^ = 14.75	<0.001
No	116 (72.50)	83 (83.00)	33 (55.00)		
Yes	44 (27.50)	17 (17.00)	27 (45.00)		
Diabetes				χ^2^ = 0.01	0.929
No	151 (94.38)	95 (95.00)	56 (93.33)		
Yes	9 (5.62)	5 (5.00)	4 (6.67)		
Cigarette smoking				χ^2^ = 0.01	0.929
No	151 (94.38)	95 (95.00)	56 (93.33)		
Yes	9 (5.62)	5 (5.00)	4 (6.67)		
Use of corticosteroids				χ^2^ = 0.08	0.781
No	118 (73.75)	73 (73.00)	45 (75.00)		
Yes	42 (26.25)	27 (27.00)	15 (25.00)		
Surgical segment				*Z* = −0.832	0.405
L2/3	2 (1.25)	1 (1.00)	1 (1.67)		
L3/4	2 (1.25)	2 (2.00)	0 (0.00)		
L4/5	114 (71.25)	68 (68.00)	46 (76.67)		
L5/S1	42 (26.25)	29 (29.00)	13 (21.67)		
BMI	23.78 ± 2.74	23.53 ± 2.33	24.19 ± 3.30	*t* = −1.36	0.175
Length of hospital stay (days)	15.00 (13.00, 17.00)	14.00 (12.75, 17.00)	15.00 (13.75, 19.00)	*Z* = −1.87	0.061
Surgical duration (minutes)	180.00 (150.00, 225.00)	182.50 (153.75, 225.00)	171.00 (150.00, 221.25)	*Z* = −0.51	0.611
Intraoperative blood loss (ml)	200.00 (150.00, 400.00)	200.00 (137.50, 400.00)	275.00 (150.00, 400.00)	*Z* = −1.10	0.270
Preoperative intervertebral space height (mm)	9.91 (8.66, 11.45)	9.96 (8.62, 11.48)	9.81 (9.01, 11.21)	*Z* = −0.02	0.982
Postoperative intervertebral space height (mm)	12.09 ± 2.22	12.18 ± 2.37	11.94 ± 1.97	*t* = 0.66	0.507
Improvement in intervertebral space height (mm)	1.89 (1.11, 3.13)	2.00 (1.11, 3.37)	1.69 (1.17, 2.82)	*Z* = −0.98	0.329
HU	119.71 ± 41.90	134.51 ± 39.76	95.04 ± 32.99	*t* = 6.47	<0.001
VBQ score	2.98 ± 0.55	2.80 ± 0.50	3.27 ± 0.50	*t* = −5.73	<0.001
PBQ score	3.15 ± 0.59	2.94 ± 0.56	3.49 ± 0.46	*t* = −6.39	<0.001

### 3.2 Multivariate logistic regression analysis

A multivariate logistic regression model was employed to identify independent predictors for cage subsidence following single-level lumbar interbody fusion surgery. The presence of cage subsidence postoperatively was set as the dependent variable, while age, hypertension, HU, VBQ score, and PBQ score were selected as independent variables. The analysis revealed that both the HU (OR = 0.98, 95% CI: 0.97–0.99; *P* = 0.018) and PBQ score (OR = 3.99, 95% CI: 1.63–9.79; *P* = 0.002) were independent predictors of cage subsidence in patients undergoing single-level lumbar interbody fusion surgery (*P* < 0.05) (Table 2).

**TABLE 2:** Multifactorial logistic regression model analysis of cage subsidence in patients after single-segment lumbar interbody fusions.

Variables	β	S.E	Wald	*P*	OR (95%CI)
Age	0.01	0.03	0.054	0.816	1.01 (0.95–1.06)
Hypertension	0.78	0.48	2.674	0.102	2.19 (0.86–5.58)
HU	−0.02	0.01	5.558	0.018	0.98 (0.97–0.99)
VBQ score	0.50	0.50	0.987	0.321	1.65 (0.62–4.40)
PBQ score	1.38	0.46	9.142	0.002	3.99 (1.63–9.79)

### 3.3 ROC curve analysis

**FIGURE 5:**
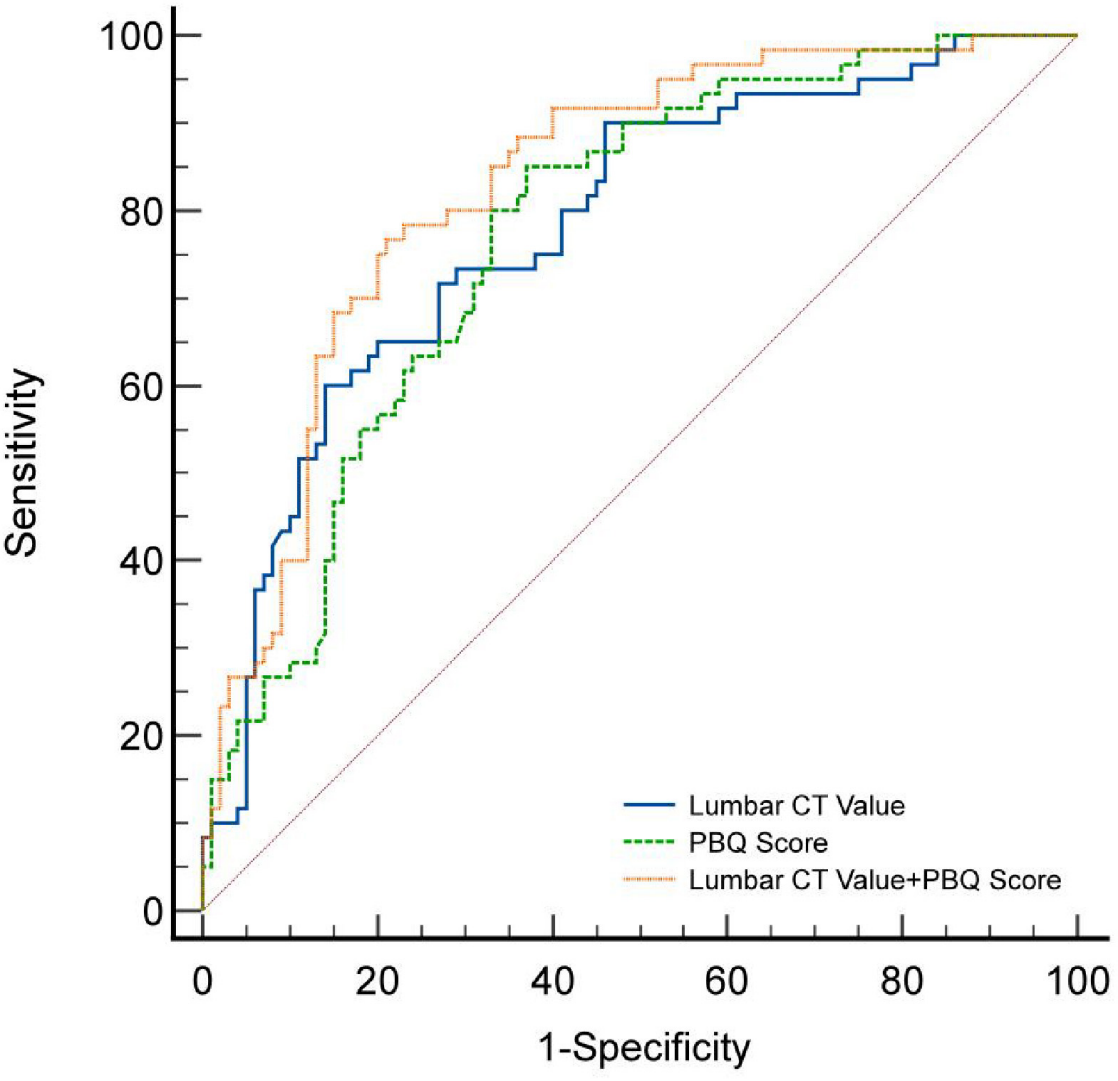
ROC curves for patients presenting with cage subsidence after single-segment lumbar interbody fusion.

We constructed Receiver Operating Characteristic (ROC) curves to assess the diagnostic efficacy of HUs and PBQ scores in predicting cage subsidence. The area under the curve (AUC) for HU was 0.781 (95% CI: 0.709–0.843; Figure 5; Table 3), with an optimal threshold of 95, a maximum Youden’s index of 0.460, corresponding to a diagnostic sensitivity of 60.0% and specificity of 86.0%. For PBQ score, the AUC was 0.772 (95% CI: 0.699–0.835; Figure 5; Table 3), with an optimal threshold of 3.088, a maximum Youden’s index of 0.480, corresponding to a diagnostic sensitivity of 85.0% and specificity of 63.0%. Additionally, the combined predictive model using both HU and PBQ score showed an AUC of 0.831 (95% CI: 0.763–0.885; Figure 5; Table 3), with a maximum Youden’s index of 0.557, corresponding to a diagnostic sensitivity of 76.7% and specificity of 79.0%. DeLong’s test results indicated that the combined diagnostic model outperformed the use of HU and PBQ score alone (*P*-values of 0.049 and 0.014, respectively).

**TABLE 3:** Sensitivity analysis of patients presenting with cage subsidence after single-segment lumbar interbody fusion.

Factor	AUC	95%CI	Sensitivity	Specificity	Youden index	Optimal threshold
HU	0.781	0.709–0.843	60.0%	86.0%	0.460	≤95
PBQ scores	0.772	0.699–0.835	85.0%	63.0%	0.480	>3.088
HU + PBQ scores	0.831	0.763–0.885	76.7%	79.0%	0.557	>0.437

### 3.4 Interobserver reliability

The interobserver reliability analysis demonstrated excellent agreement between the two radiologists for all primary radiological measurements. The ICC for the PBQ score was 0.91 (95% CI: 0.87–0.94), for the VBQ score was 0.89 (95% CI: 0.84–0.92), for the HU was 0.93 (95% CI: 0.90–0.95), and for the intervertebral disc height was 0.88 (95% CI: 0.83–0.92).

## 4 Discussion

The purpose of this study was to evaluate the predictive value of pedicle bone quality score (PBQ score) and HUs for cage subsidence after single-level lumbar fusion surgery. The results indicate that both HUs and PBQ scores are significant predictors of cage subsidence after single-level lumbar interbody fusion, with high predictive ability. Moreover, their combined use significantly improved the accuracy of prediction. These findings provide important reference points for clinicians in preoperative risk assessment and the development of personalized treatment plans.

HU is an important indicator for assessing vertebral bone quality (14–16) and plays a significant role in predicting the risk of cage subsidence after single-level lumbar fusion surgery. In this study, the area under the curve (AUC) for HU was 0.781, with an optimal threshold of 95, diagnostic sensitivity of 60.0%, and specificity of 86.0%. This suggests that when a patient’s preoperative HU is below 95, clinicians should maintain a high level of suspicion for the risk of postoperative cage subsidence and consider optimizing the surgical plan. Previous studies have also shown that HUs are of high value in predicting cage subsidence after lumbar fusion, with AUC values similar to those found in this study (17–19). HUs reflect vertebral bone density, and lower HUs often indicate osteopenia or fragility of the bone structure, which can lead to insufficient support for the fusion cage, thereby increasing the risk of subsidence (20). Furthermore, HUs provide morphological information about the integrity of the bone structure. Decreased trabecular bone and thinning of cortical bone reduce the vertebral body’s mechanical strength (21), further affecting load distribution and leading to localized stress concentration. The postoperative bone healing process relies on the quality of the surrounding bone tissue, and patients with lower HUs may face a higher risk of poor healing, increasing the likelihood of cage subsidence (22, 23). Therefore, routine preoperative measurement of HU provides a theoretical basis for optimizing surgical plans and reducing postoperative complications. This approach not only helps improve surgical success rates but also enhances postoperative outcomes.

In this study, we proposed a novel pedicle bone quality (PBQ) score, which is measured based on the previously described vertebral bone quality (VBQ) score, but using magnetic resonance imaging (MRI). The PBQ score quantifies the bone density and morphological characteristics of the pedicle region, effectively reflecting the bone quality in this area (5, 8). The results of this study indicate that PBQ score has a significant impact on the occurrence of cage subsidence after single-level lumbar fusion surgery. Furthermore, the multivariate regression results showed that PBQ score (OR = 3.99, 95% CI: 1.63–9.79; *P* = 0.002) is an independent risk factor for cage subsidence after single-level lumbar interbody fusion, with an increased risk of subsidence as the PBQ score increases. The area under the curve (AUC) for PBQ score in predicting cage subsidence after single-level lumbar fusion was 0.772, with an optimal threshold of 3.088, diagnostic sensitivity of 85.0%, and specificity of 63.0%, demonstrating high predictive value.

There is a close relationship between PBQ score and pedicle bone mass. When the PBQ score is high, the pedicle bone mass of the patient may be low, which could result in insufficient support for the pedicle screw-rod system, thus increasing the risk of cage subsidence (24). Compared to the VBQ score, the PBQ score provides a more detailed analysis of bone quality in the pedicle region. Our findings also suggest that PBQ score has a more significant impact on the occurrence of cage subsidence after lumbar fusion compared to the VBQ score, and the predictive value of PBQ score alone for postoperative cage subsidence is roughly comparable to that of HU. Additionally, compared to previous studies, the AUC value for PBQ score in predicting cage subsidence after lumbar fusion is similar to the reported AUC values for VBQ (25–27). This further confirms the importance of PBQ score in predicting cage subsidence after lumbar fusion surgery.

The combined use of HU and PBQ score demonstrated a significant advantage in predicting cage subsidence after single-level lumbar fusion surgery. The area under the curve (AUC) of the combined model was 0.831, with a maximum Youden index of 0.557, diagnostic sensitivity of 76.7%, and specificity of 79.0%. This result suggests that combining these two indicators allows for a more comprehensive assessment of the patient’s bone quality and provides a more accurate prediction of cage subsidence risk. This finding is consistent with the study by He et al., who pointed out that combining multiple imaging parameters can significantly improve the prediction of postoperative complications (28). Furthermore, the assessment of PBQ score and HU is both readily available and highly reproducible in routine clinical practice, with relatively low associated costs. Therefore, preoperative measurement of PBQ score and HU should be used to evaluate both the overall and local bone quality of the vertebrae. The combined model can more comprehensively assess the patient’s risk and assist surgeons in developing a more reasonable surgical plan.

Furthermore, the excellent interobserver reliability (ICC > 0.9) of the PBQ score established in this study confirms its reproducibility as a measurement tool. This high level of agreement between human observers is a critical first step toward future automation. The development of artificial intelligence (AI) algorithms to automate PBQ and HU measurement is a promising and logical next step. Such automation could further enhance the scalability, speed, and standardization of these assessments, facilitating their integration into routine clinical workflow for preoperative planning.

This study has limitations. Its retrospective, single-center design may introduce bias and limit its generalizability. While the sample size is adequate, it is modest, warranting larger, multi-center validation in future. The 18-month follow-up was sufficient for detecting early subsidence but is too short to assess long-term outcomes such as fusion rates or adjacent segment degeneration. Furthermore, we focused on radiographic subsidence and did not correlate its degree with patient-reported symptoms. Additionally, the 2D MRI-based PBQ score lacks a formal analysis of variability across pedicle sections. Other confounders, such as cage design and surgical technique, were not controlled for. Lastly, the study did not directly compare the PBQ score with DXA BMD T-scores, nor did it systematically analyze endplate morphology. These are important factors that future research should incorporate.

## 5 Conclusion

This study confirms the importance of PBQ score and HU in predicting cage subsidence after single-level lumbar interbody fusion. The predictive model constructed by the combined evaluation of these two factors showed good diagnostic performance (AUC = 0.831), providing clinicians with a more accurate risk assessment tool. This helps identify high-risk patients preoperatively, optimize treatment plans, and thereby improve the success rate of single-level lumbar fusion surgery and the postoperative prognosis of patients.

## Data Availability

The original contributions presented in this study are included in this article/supplementary material, further inquiries can be directed to the corresponding authors.
